# Impaired early information processing in adult ADHD: a high-density ERP study

**DOI:** 10.1186/s12888-020-02706-w

**Published:** 2020-06-10

**Authors:** Szilvia Papp, László Tombor, Brigitta Kakuszi, Lívia Balogh, János M. Réthelyi, István Bitter, Pál Czobor

**Affiliations:** Department of Psychiatry and Psychotherapy, Semmelweis University, HU1083, Balassa utca 6., Budapest, Hungary

**Keywords:** ADHD, Early sensory deficit, ERP, Sensory modulation, Sensory processing difficulties

## Abstract

**Background:**

Children with attention-deficit/hyperactivity disorder (ADHD) often demonstrate sensory processing difficulties in the form of altered sensory modulation, which may contribute to their symptomatology. Our objective was to investigate the neurophysiological correlates of sensory processing deficits and the electrophysiological characteristics of early information processing in adult ADHD, measured by the P1 event-related potential (ERP).

**Methods:**

We obtained ERPs during a Go/NoGo task from 26 adult patients with ADHD and 25 matched controls using a high-density 128-channel BioSemi ActiveTwo recording system.

**Results:**

ADHD patients had a significantly reduced P1 component at occipital and inferotemporal scalp areas compared to controls. The reduction was associated with inattention and hyperactivity symptom severity, as measured by the Conners’ Adult ADHD Rating Scale. ADHD patients with higher inattention scores had significantly smaller P1 amplitudes at posterior scalp sites, while higher hyperactivity scores were associated with higher P1 amplitudes.

**Conclusions:**

Deficits in early sensory processing, as measured by the P1 ERP component, are present in adult ADHD patients and are associated with symptom severity. These findings are suggestive of bottom-up cognitive deficits in ADHD driven by impairments in early visual processing, and provide evidence that sensory processing problems are present at the neurophysiological level in this population.

## Background

Attention-deficit/hyperactive disorder (ADHD) affects around 3–6% of children [1] and persists into adulthood at a prevalence of 2.5% [2]. On the symptomatic level, ADHD is characterized primarily by inattention, hyperactivity and impulsivity [3]. While ADHD symptoms and their consequences were thought to diminish when reaching adulthood, deficits are often persistent [4], leading to impaired health-related quality of life of adult ADHD patients [5, 6].

Research of childhood ADHD suggests [7] that besides these core symptoms, ADHD may also affect sensory processing (i.e., the ability of the central nervous system to collect, process and organize responses to sensory information) and sensory modulation (the ability to regulate the degree, intensity and nature of responses to sensory input) [8, 9], in the form of higher sensory dysfunction (measured by both rating scales and by physiological reactions to stimuli [7, 10]. Sensory processing problems are severe enough in every sixth child with ADHD to have a negative impact on their everyday life [11]. Even though difficulties arising from altered sensory modulation are believed to adversely affect core ADHD symptomatology, the distinct pattern of sensory processing deficits in ADHD patients is yet to be described [12].

To gain insight to the neural mechanisms associated with symptoms of psychiatric disorders, including ADHD, electro-encephalography (EEG) and event-related potentials (ERPs) are commonly employed. Due to its high temporal, and - with high-density EEG – good spatial resolution, EEG offers a detailed understanding of specific cognitive dysfunctions.

While current research focuses mainly on the later timeframe of information processing, the presence of ADHD is thought to affect not only the top-down attentional selection but also the early, bottom-up sensory processing [13, 14]. Generated in extrastriate visual areas [15], ERP components P1 and N1 represent the early stages of perceptual processing. The P1 component (latency 80-150 ms) has traditionally been associated with basic visual processing and spatial attention, along with data supporting the ‘sensory gain control/amplification’ hypothesis that attention directed to the location of the stimulus (inside the receptive field) is associated with increased amplitudes of the P1 and N1 components [16, 17]. Electrophysiological evidence to the ‘capacity theory’ supports hypotheses which connect perceptual load to early spatial selection in visual processing, namely that spatial selection is directly dependent on perceptual load [18].

In case of children with ADHD a reduction in the P1 component has been demonstrated using a wide range of paradigms including oddball [19–21], stop-signal [22] and CPT (continuous performance task) tasks [23, 24]. In their visual cued CPT study of children with ADHD, Nazari et al. [24] demonstrated a decreased P1 amplitude in ADHD patients in the NoGo condition as compared to their control peers, suggesting an early deficit in visual sensory integration. EEG data were collected from 64 surface electrodes, peak amplitudes for P1 were measured at 3 occipital electrode sites. The source of P1 was localized to the occipital area by the swLORETA (standardized weighted low resolution electromagnetic tomography) method. Nonetheless, the results overall remain controversial, with some of the research groups [25–28] finding insignificant differences between (childhood) ADHD and control groups regarding the P1.

In the adult ADHD literature, Woltering et al. [29] in their dense array electroencephalography Go/NoGo study of 54 college students with ADHD and their typically developing peers did not find group differences between patient and control groups regarding the P1 component. This finding could be attributed to the relatively low perceptual processing demands of the paradigm, and may not generalize to other, more demanding tasks [29]. In a 64-electrode EEG setting, Raz and Dan [30] found higher P1 amplitude in the adult ADHD group at occipital and posterior-parietal scalp locations in response to both happy and angry faces, which may reflect specific hyper-vigilance in the visual cortex of adult ADHD patients to emotional faces. However, in the neutral emotional valence setting (response to neutral faces), no such difference was found between patient and control groups. Taking these results together, in the present study we focused on the NoGo trials as we wanted to increase the perceptual load and thereby the assay sensitivity for detecting a group-difference, because we expected that the basic visual processing and early spatial selection is more impaired when the perceptual load is high (as during the NoGo trials in a Go/NoGo task, where the prepotent response tendency is high).

With high-density EEG, due to its excellent time-resolution and topographical information on the whole scalp area, it is possible to gain a more detailed insight into the early sensory (‘bottom-up’) processes. In the current study, in addition to focusing on the region of interest electrodes that were predominantly examined in prior studies, our main goal was to conduct a detailed topographical analysis of the early processing of the sensory information in adult ADHD patients and healthy controls based on high density (128-channel) EEG recordings. Previous research which examined the topographical distribution of early ERP components in ADHD patients either relied on sparse spatial sampling, or used a lower number of electrodes. However, as shown by Srinivasan et al. [31], ‘ … there does not appear to be a clear asymptote in the scalp topography when the 128-channel data are compared with 64-channel recordings. Instead, there are many examples in which the 128-channel maps show a striking in-crease in topographic detail.’ Additionally, as indicated by the studies of Song et al. [32] and Freeman et al. [33], accuracy of EEG spatial frequency representation over the scalp can be substantially augmented by increasing the number of electrodes from 64 to 128.

Given the limited and somewhat controversial and methodologically diverse ERP data on adult ADHD, this study with its Go/NoGo paradigm previously successfully used in functional imaging studies [34, 35] aims to provide a detailed investigation of the early sensory stages of information processing reflected in the P1 component. Based on previous literature of childhood ADHD [19–24] we expected that P1 would be affected in adults with ADHD as well, with a lower observed P1 amplitude in ADHD patients.

## Methods

### Participants

Fifty-one subjects participated in the study, including 26 adult ADHD patients and 25 healthy control subjects. Healthy controls were matched to the patients on age (±5 years), gender and level of education. Control subjects were excluded in case of neurological or psychiatric history. In control subjects, the absence of a current psychiatric condition was confirmed with the Symptom Checklist 90R (SCL-90R) [36]. No control subjects were excluded based on SCL-90R scores. Patients participating in the study were diagnosed with ADHD persisting into adulthood by an experienced psychiatrist based on a detailed clinical interview. The interview consisted of the following steps: structured interview for assessing current and (retrospective) childhood DSM-IV-TR ADHD symptoms; semi-structured and open interviews assessing background information, developmental data, functional impairment, psychiatric comorbidity; medical history data obtained from medical documentation and close family members; and finally, a wide range of self-rated questionnaires including the Conners’ Adult ADHD Rating Scale – Self-Report: Long version (CAARS; 66-item, self-reported and long version [37]. All patients met the DSM-IV-TR diagnostic criteria of adult ADHD (16 combined subtype, 3 hyperactive subtype, 7 inattentive subtype) [38]. A subset of patients (*n* = 9) was on methylphenidate medication monotherapy, three patients were receiving bupropion (one on monotherapy, one on antidepressant combination, one combined with methylphenidate) while fourteen patients were medication-naive. ADHD patients on stimulant medication were required to go off their medication 24 h prior the EEG session. The study complied with the ethical standards of the Declaration of Helsinki, and received approval from the Ethical Committee of Semmelweis University. All participants gave written Informed Consent for the study.

### Stimuli and procedure

Participants were seated in a dimly lit room; a computer screen was placed at a viewing distance of approximately 50 cm. The applied Go/NoGo paradigm was previously used and described by Durston et al. [34]. In accordance with the original study, characters from the Pokemon cartoon series were used as visual stimuli. The experiment was programmed and presented with the Presentation 13.0 software (Neurobehavioral Systems, Inc.). Participants were instructed to respond as quickly and accurately as possible by pressing a button upon the appearance of Go trials; they were, however, asked to withhold response in case of rare NoGo trials. The task consisted of 5 runs. Pictures were presented for 1 s with an interstimulus interval of 3 s. Runs consisted of 57 pictures, of which 75% were Go trials, and 25% were NoGo trials. During the task, different types of NoGo trials were presented, NoGos were preceded either by 1, 3, or 5 Go trials. Foil trials (NoGo trials after 2 or 4 Go trials) were also administered to prevent learning; however, those were not included in the analysis. The different types of NoGo trials were pseudorandomly presented.

### Clinical measures

ADHD symptom severity was described by the Conners’ Adult ADHD Rating Scale-Self-Report: Long Version (66-item, self-reported version) across core psychopathological domains of ADHD: inattention, hyperactivity, impulsivity and problems with self-concept [37]. The total score on the SCL-90R was used to measure the severity on general domains of psychopathology. Based on the original criteria, a global severity index of > 114 on the SCL-90R was considered high risk for a psychiatric disorder [36, 39].

### EEG recording and preprocessing

EEG was recorded using a 128-channel active electrode system (BioSemi ActiveTwo). The electrode cap covered the whole head with an equidistant-layout. Eye movements were monitored by two electrooculogram (EOG) electrodes placed below the left and above the right external canthi. Data were digitized at a sampling rate of 1024 Hz, with a band-pass filter of 0.5-70 Hz using the BioSemi recording system with average reference. Data were stored and analysed off-line subsequently using the Electromagnetic Source Signal Imaging (EMSE) Suite as well as the Statistical Analysis System (SAS 9.4) software. Artefact rejection, including the exclusion of eye movement artefacts based on horizontal and vertical EOG, and subsequently the exclusion of epochs with a voltage exceeding ±90 μV on any EEG or EOG channel was conducted applying automatic artefact rejection criteria. For stimulus-locked ERP, epochs of 900 ms duration from 100 ms prestimulus to 800 ms poststimulus were extracted from the continuous EEG. The -100 ms timepoint was selected in order to establish pre-stimulus baseline, while the 800 ms post-stimulus timepoint was chosen to cover most of the stimulus presentation period, ending before its offset. Our selected timeframe for the ERP epochs is similar to that used in the literature by others who investigated ERP responses in adult ADHD patients (e.g.: Raz and Dan [30] or Helenius et al. [40]). Altogether, the number of analysed epochs for ADHD patients and controls were 260 and 276, respectively.

### ERP analysis and behavioural measures

ERPs during correct NoGo conditions were recorded and averaged at all 128 electrode sites. Time windows for hypothesized components were determined a priori. Specifically, the P1 was defined as the mean amplitude deflection (i.e., area under curve) occurring in the window from 120 to 150 ms post-stimulus [41]. When investigating relationships between ERPs and clinical measures, midline electrodes (Fz, Cz, Pz and Oz) as sites of interests were defined based on a review of the literature of Go/NoGo tasks used in adult ADHD studies [29, 40, 42, 43].

Since early visual ERP components such as P1 typically exhibit polarity reversal in the posterior-to-anterior direction [44], at the midline electrode sites of interest we expected that the P1 amplitude will change along the sagittal axis from positive (occipitally) to negative sign (frontally).

Performance was assessed with measures of the mean reaction time and commission error rates. For reaction times, large values that exceeded the upper threshold of 1 s and small values that were below the threshold of 250 msec (and were therefore unlikely to be stimulus-locked responses) were excluded from the analyses. Therefore, the accepted reaction time range was between 250 and 1000 ms (inclusive). Less than 0.5% of the individual reaction time values were rejected in each of the two groups on the basis of these threshold criteria (0.39 and 0.42% in the control and patient group, respectively). We checked the distribution of the reaction times after excluding values that were outside the accepted range. As indicated by the Kolmogorov-Smirnov D-statistic, the distribution of reaction times deviated from the normal distribution (*p* < 0.01 in each of the two study groups). We also investigated the distribution of error rates, and found that it - similarly to reaction times - deviated from the normal distribution in both study groups (p < 0.01). Since the distribution of reaction times and error rates was right skewed with increasingly higher values occurring with increasingly lower frequency, these behavioural data were analysed by applying the Generalized Linear Mixed Model (GLIMMIX) analysis with a logarithmic link function to compare the two study groups. This approach makes allowance for non-normally distributed data, such as the right skewed distribution of behavioural data in our study. Measure of central tendency for the reaction time and commission error rate in each group was characterized by the mean and 95% confidence limits, which were derived as backtransformed data from the GLIMMIX procedure from logarithmic to the original units (msec or error rate).

### Statistical analyses

The primary statistical analysis for group difference between ADHD and control subjects was based on the random regression hierarchical linear model (HLM). Amplitude (voltage) values within the time-window of interest (120-150 ms) were used as dependent variable in the HLM. Group, time (sampling point) and their interaction were applied as independent variables; age and gender and level of education served as covariates in all analyses. A separate analysis was performed for each scalp site of interest (all 128 electrodes, including Fz, FCz, Cz, Pz and Oz). For these topographical analyses using the full set of the 128 channels, the False Discovery Rate (FDR)-corrected *p*-values [45] were computed. The alpha-level of 0.05 (adjusted for multiple comparisons) was adopted for statistical significance.

In order to delineate the topographical distribution of the differences, we investigated whether the EEG recording from multiple individual channels aggregated into electrode clusters with respect to group differences. We defined electrode clusters as a group of at least five adjacent scalp derivations with significant group difference in the same direction.

For scalp sites of interests that yielded a significant group difference in the primary analysis after Bonferroni correction for multiple testing, we conducted additional analyses to test whether psychopathological variables served as covariates in explaining the significant alterations in early sensory activity. These covariates included the total score on the CAARS Hyperactivity, Impulsivity, Inattention, and Problems with Self-Concept domains. In subsidiary analyses, we also investigated whether comorbidity (present/absent) and medication status (stimulant treatment yes/no; any psychopharmacological treatment yes/no) impacted our results. In these analyses, the latter variables were included as additional covariates in the HLM model.

## Results

### Demographics and basic descriptive characteristics

Basic demographic and clinical characteristics of the study population are provided in Table 1.

**Table 1:** Basic demographic and clinical characteristics of the study sample^a^

Characteristics	Control (*N* = 25)	ADHD (*N* = 26)	Chi^2^	P
Male, N (%)	19 (76.0)	18 (69.2)	0.29	0.76
Medication				
Methylphenidate, N (%)	–	9 (34.6)	n/a	n/a
Antidepressant, N (%)	–	2 (7.69)	n/a	n/a
Anxiolytic, N (%)	–	1 (3.85)	n/a	n/a
Comorbidity				
Depressive disorder, N (%)	–	4 (15.38)	n/a	n/a
Anxiety disorder, N (%)	–	4 (15.38)	n/a	n/a
Both, N (%)	–	3 (11.54)	n/a	n/a
			F	P
Mean age, mean (SD)	27.3 (5.0)	28.9 (8.4)	0.71	0.40
Years of education, mean (SD)	16.3 (1.6)	14.0 (2.5)	13.94	0.0005
CAARS^b^				
Inattention, mean (SD)	11.1 (7.9)	23.7 (7.0)	32.09	< 0.0001
Hyperactivity, mean (SD)	10.1 (6.3)	20.1 (4.8)	36.74	< 0.0001
Impulsivity, mean (SD)	9.1 (6.5)	17.6 (7.4)	16.98	0.0002
Problems with Self Concept (SD)	4.8 (4.4)	10.1 (5.5)	12.92	0.0008
SCL-90R^c^, mean (SD)	33.1 (30.1)	86.3 (51.3)	16.48	0.0002
Behavioural measures				
Commission errors, %, mean (95% confidence limits)	3.04 (2.32–3.98)	8.85 (7.63–10.2)	46.33	< 0.0001
Reaction time, msec, mean (95% confidence limits)	508.5 (480.12–538.55)	502.51 (475.27–531.31)	0.09	0.7670

^a^Chi-square test for categorical, Analysis of Variance for continuous variables. Generalized Linear Mixed Model (GLIMMIX) analysis was applied for the behavioural measures since this approach makes allowance for non-normally distributed data; mean and 95% confidence limits were estimated from the GLIMMIX model

^b^CAARS = Conners’ Adult ADHD Rating Scales

^c^SCL-90R = Symptom Checklist-90R

The control and the ADHD group did not differ in main demographic variables, including age and gender. As shown by Table 1, the patient group had a somewhat lower achievement in terms of years of education (by < 3 years); the difference obtained statistical significance. The ADHD group had higher scores on the SCL-90R scale measuring general psychopathology, and was characterised by higher severity on all specific symptom dimensions, including the CAARS factors of Inattention, Hyperactivity, Impulsivity and Problems with Self-Concept. A total of 11 (42.3%) of 26 patients had comorbidity according to the DSM-IV system. All comorbidities fell into DSM-IV affective-categories, including depressive (*n* = 4), and anxiety disorders (n = 4) or both (*n* = 3). Approximately half of the 26 patients had psychopharmacological treatment (*n* = 12, [46.2%]); these included patients who received methylphenidate (*n* = 9, [34.6%]), and 3 patients who had antidepressants (*n* = 2) or anxiolytic (n = 1).

### Behavioural outcomes

Behavioural data including reaction times and accuracy was collected by the Presentation software during task performance. Incorrect NoGo responses represented commission errors, while incorrect Go responses omission errors. Reactions times and accuracy were compared between the two groups (ADHD vs. control subjects) using the analysis of covariance (ANCOVA) procedure controlling for age, gender and education as implemented in SAS software version 9.4 (SAS Institute Inc., Cary, NC, USA). Behavioural data are summarized in Table 1. Mean reaction time was lower in ADHD patients than in control subjects, but the difference did not reach the level of significance (502.51 ms vs. 508.5 ms, *p* = 0.7670). Overall, ADHD patients were less accurate on commission (false NoGo) trials (*p* < 0.001) than on omission trials. Commission error response rates were on average 8.85% in ADHD, and 3.04% in control subjects (Table 1).

### ERP

**Fig. 1:**
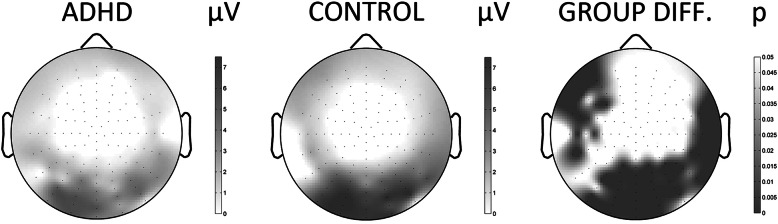
Topographical maps of the P1 component based on the full set of 128 individual channels for the NoGo condition in ADHD patients and in control subjects. The scalp maps were generated on the basis of the average voltage values in the time-window of the P1 component. Clusters of at least five adjacent scalp derivations with significant group difference in the same direction were defined as electrode clusters, depicted here. Black-and-white coding represents the amplitude value in microvolts, with darker colours corresponding to higher amplitudes. False discovery rate (FDR)-corrected map of Type-I error-probabilities for the group-difference of raw amplitude values Control-ADHD (μV) where darker shades represent larger group differences

To demonstrate the scalp-distribution of ERPs, Fig. 1 depicts the topographical map of the group differences in the 120-150 ms time-window. Besides the topographical map of ERP-voltages, the FDR-corrected map of Type-I error-probabilities is provided. Differences between patient and control groups regarding ERP amplitudes were significant at several brain regions and retained significance after correction for multiple testing. Specifically, ADHD patients showed significantly reduced P1 amplitude at occipital and inferior-temporal areas compared to controls in the NoGo condition *(*Table 2*).* Besides Type-I error-probabilities the table provides the covariance adjusted Least-Squares Mean (LSMean) estimates of the ERP amplitudes for both the ADHD and the control group.

**Table 2:** Control vs. ADHD: group differences^a^ in the P1 component in the midline 10–20 electrodes^b^

CHANNEL	GROUP (μv, SE)		F	P
	ADHD^c^	CONTROL^c^		
**Fz**	−0.87 (0.09)	−0.83 (0.09)	0.07	~ 1.0
**FCz**	−1.20 (0.09)	−1.63 (0.09)	11.82	0.1541
**Cz**	−0.85 (0.06)	−1.61 (0.06)	71.88	<.0001
**Pz**	0.94 (0.09)	1.49 (0.09)	18.57	0.0101
**Oz**	4.12 (0.15)	6.32 (0.15)	104.45	<.0001

^a^ Random Regression Hierarchical Linear Model analysis with group, time and interaction as independent variables, and with age, gender and years of education as covariates

^b^Time window for the P1 component: 120-150 ms poststimulus

^c^ Least-squares means estimates (SE) of stimulus-locked ERP amplitudes for a given study group, adjusted for age, gender and years of education

Since in the adult ADHD EEG literature midline electrodes traditionally have been used as electrode sites of interest where ERP amplitudes are investigated in more detail, besides our ‘whole brain’ approach, we also focused on ERP analyses at Fz, FCz, Cz, Pz and Oz 10–20 midline electrodes. Considering that our results showed that ADHD patients have significantly reduced NoGo P1 amplitude at occipital and inferior-temporal areas including Cz, Pz and Oz electrodes sites, focusing on midline electrode sites enabled comparison of our results with the literature.

**Fig. 2:**
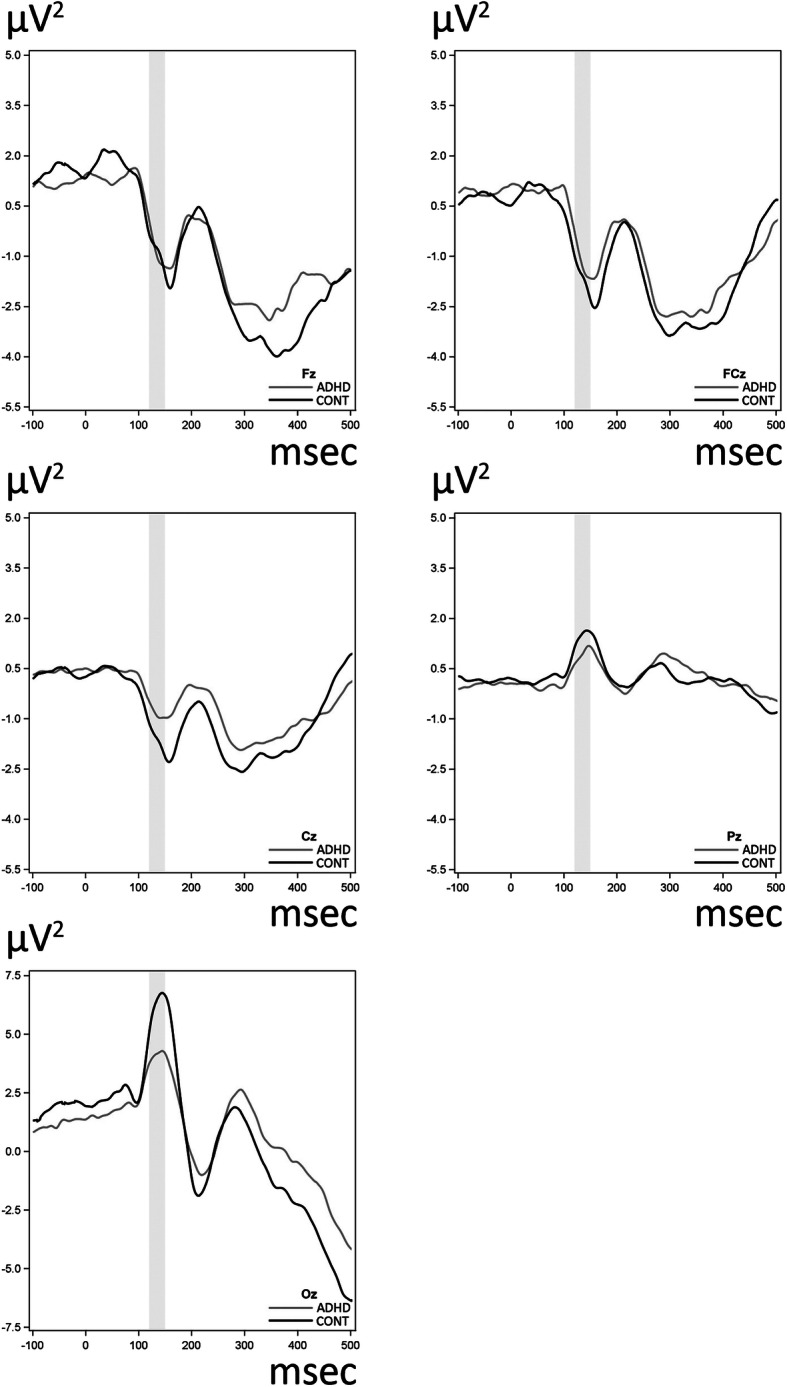
Waveforms for raw amplitude (μV) values for stimulus-locked ERPs in five typical scalp sites (Fz, FCz, Cz, Pz, Oz). The waveforms are displayed for both groups for the NoGo condition (commission-error responses). Time-window for the P1 ERP component is shaded

Figure 2 shows the grand average ERPs of the midline electrodes for NoGo stimuli for the ADHD and the control groups. As for the 10–20 midline electrodes, a significant difference was found between the ADHD and the control group including and surrounding the Cz, Pz and Oz sites in the 120-150 ms timeframe with lower ERP amplitudes in the patients. We note that in our subsidiary analyses we examined whether the aforementioned group differences are present after adjustment for comorbidity and medication status. These analyses showed that all results which were statistically significant retained their significance after including comorbidity and medication status as additional covariates in the analyses.

### Relationship between ERP and clinical characteristics

Dimensional associations between the P1 ERP component and psychopathological variables, including the severity on the CAARS Hyperactivity, Impulsivity and Inattention subscales were examined using the data of all participants combined. The analysis was conducted for those midline electrodes where a significant group-difference was present in the P1 time-window. Based on the HLM model, we determined the LSMeans for the P1 ERP amplitude for low and high severity of CAARS Hyperactivity, Impulsivity and Inattention subscales in order to interpret the direction of the associations.

After corrections for multiple comparisons, we found that the Inattention factor was related to ERP changes at Cz, Pz and Oz electrode sites (F = 1.45, *p* < .0001; F = 58.56, p < .0001; F = 17.74 *p* = 0.0001, respectively). Investigation of the direction of the relationship revealed smaller P1 amplitudes among those subjects who had higher severity on Inattention as compared to subjects with lower subscale severity. Hyperactivity scores were also associated with ERP changes at Cz and Pz sites (F = 4.54, *p* = 0.04; F = 11.71 *p* = 0.0014, respectively), with the opposite direction: patients with higher hyperactivity scores had significantly higher P1 amplitudes. The relationship between altered early ERP activity and CAARS symptom dimensions is shown in Table 3.

**Table 3:** Relations between symptom severity as measured by CAARS and P1 ERP amplitudes on midline electrode sites between 120 and 150 ms.

CHANNEL	CAARS DOMAIN	SYMPTOM SEVERITY (μV, SE)		DIFFERENCE F (P)^a^
		LOW	HIGH	
**Cz**	HYPERACTIVITY	−0.37 (0.14)	0.05 (0.17)	4.54 (0.04)
	IMPULSIVITY	−0.41 (0.10)	−0.29 (0.18)	0.53 (0.47)
	INATTENTION	−0.91 (0.14)	−0.37 (0.14)	1.45 (<.0001)
**Pz**	HYPERACTIVITY	−0.35 (0.22)	0.75 (0.28)	11.71 (0.0014)
	IMPULSIVITY	0.52 (0.17)	0.01 (0.29)	4.23 (0.05)
	INATTENTION	1.57 (0.21)	0.19 (0.21)	58.56 (<.0001)
**Oz**	HYPERACTIVITY	2.78 (0.36)	2.56 (0.45)	0.19 (0.6674)
	IMPULSIVITY	2.36 (0.27)	1.58 (0.46)	3.77 (0.0590)
	INATTENTION	1.65 (0.35)	0.67 (0.35)	17.74 (0.0001)

^a^corrections for multiple comparisons were applied

## Discussion

The current study investigated the behavioural and neurophysiological correlates of early visual processing reflected in the P1 ERP component in a visual Go/NoGo task in a cohort of adults with ADHD and their healthy controls.

Despite existing controversies regarding their task performance, patients with ADHD often perform poorer in the laboratory setting than control subjects on Go/NoGo inhibition tasks [29, 46, 47]. In our Go/NoGo paradigm [35], we used a long interstimulus interval, which could explain the observed high performance of both groups regarding commission errors. Despite this finding, however, in lines with the literature, patients with ADHD made significantly more commission errors than controls in our study.

As for reaction times, we found a rather modest, non-significant difference between patients and control subjects, with patients being faster than controls. This finding is consistent with a significant number of previous observations of childhood ADHD [28, 48], and is also in line with some ADHD data reporting adult patients not being significantly different from healthy controls in terms of reaction time [29, 43, 46, 49].

The limited number of ERP studies on early information processing provided inconsistent results (for a review see Johnstone et al. [50]), with some reporting altered P1 amplitudes in adult ADHD subjects compared to controls [30] and others failing to confirm this [29]. It is important to note that the EEG methodology in the literature is diverse. In our study a high density EEG setting provided data on the whole brain area, while a long interstimulus interval assured that the repetition of stimuli did not confound early sensory components. As for further methodological issues, while some of the above studies have used a dense array EEG (e.g. Woltering et al.– 64 channels [29], Raz and Dan – 64 channels [30]), ERP peak amplitudes are usually measured at a lower number of traditionally used electrode sites.

The main finding of our EEG study is that adults with ADHD showed a reduced P1 amplitude. The P1 is the first positive ERP component present around 100 ms after stimulus, associated with basic visual processing and spatial attention. ERP amplitude is thought to reflect the activation level of the activated central nervous system neuronal population [24]. Therefore, the reduced amplitude of the P1 component is considered to reflect an early sensory deficit present in adults with ADHD.

While in the ADHD literature later ERP components are more commonly studied, in schizophrenia research the P1 component is considered an endophenotype measure [51] with consistent deficits in the early visual processing in patients with schizophrenia as indexed by a reduced P1 component. This reduction of the P1 amplitude, observed as well in our sample of patients with ADHD can be related either to a limited capacity in focusing attention or to a decreased attentional gain control mechanism on early sensory responses [24, 48]. Prox et al. [43] suggest that differences in later ERP components indicate that ADHD patients may be overcoming the described early sensory deficits by more effortful later-stage processing. In lines with this, Donohoe et al. [51] state that the P1 is indeed ‘cognitively penetrable’ with early stages of information processing appearing to be reciprocally modulated by higher processing areas (top-down effects). Therefore, it could be argued that in patients with ADHD early sensory processing deficits may contribute to susceptibility to distraction/inattention in the illness, with top-down control aiming to adjust this impact on ADHD symptoms (i.e., inattention and impulsivity).

Based on both parent-reported measures and psychological assessment, sensory profile of children significantly differs from their typically developing peers, including the visual domain [52]. These difficulties have been analysed in some studies with both behavioural and neuropsychological measures [9]. With the high temporal resolution of EEG, we were able to observe early sensory processing in a reliable, detailed manner. Therefore, altered early sensory ERPs, including P1 could provide further, electrophysiological evidence of altered sensory processing in patients with ADHD.

The above mentioned P1 amplitude alterations were not only present on occipital regions (the activity of which primarily reflects electrophysiological changes of sensory brain areas), but we also found significant differences between ADHD and control groups regarding the P1 ERP amplitude on left inferior-frontal and inferotemporal EEG channels. Prior studies found that the left inferior frontal gyrus (IFG) plays a crucial role in top-down control, and is reciprocally connected to more posterior regions, including lateral temporal cortices. For example, in a phonological test using functional magnetic resonance imaging (fMRI), Bitan et al. [53] examined top-down control among various brain regions, and found that the left IFG elicits a selective enhancement of task-specific processing in posterior brain regions. Our findings of decreased P1 amplitude in this region are therefore also consistent with the idea of a weakened top-down control in ADHD for the processing of task relevant versus task-irrelevant information [54], which may be due to neurodevelopmental changes that were described in ADHD, especially in the frontal areas [35, 55].

Our second goal was to investigate whether alterations in the early sensory processing stages in ADHD are related to psychopathological symptoms. We found that the P1 amplitude reduction correlated with symptoms of inattention as measured by the Conners’ Adult ADHD Rating Scale [37]. This association indicated that our subjects with higher inattention scores showed more pronounced electrophysiological changes at posterior scalp sites. On the other hand, a different pattern was observed regarding the connection between CAARS Hyperactivity subscale and P1 amplitude; lower amplitudes were associated with lower symptom severity as measured by the CAARS Hyperactivity subscale.

In particular, the P1 amplitude alteration was associated with higher symptom severity on the CAARS scale regarding inattention at central/posterior electrode sites, while patients with higher hyperactivity scores had a higher (less attenuated) P1 component. These findings are consistent with the idea that symptom presentation in ADHD may not be a unitary phenomenon, and may reflect multiple aetiologies. They are also in line with the causal model of Sokolova et al. [56], which considers inattention a driving factor for hyperactivity/impulsivity, whereas those factors which lead to high hyperactivity/impulsivity do not necessarily lead to higher inattention. Also, inattention can be viewed in the context of a possible sensory deficit. In this context, poor inattention may be modulated by higher hyperactivity through a pathological compensatory gain control mechanism.

Although ERP research has grown extensively in the past decades, besides a need for a more rigorous approach to increase the consistency and generalizability of electrophysiological findings [50], it is noteworthy that studies generally rely on analysis of traditional single channel waveforms (typically in Fz, Cz, and Pz areas). Extending this focus and providing high-density maps of topographical distribution would result in a statistically more confident delineation and a better understanding of electrophysiological changes as well. Dysfunctional early stages of information processing can result in deficits in later stages as well, therefore, we suggest that further research efforts should address these alterations more explicitly. Another important aspect regarding further studies would be a more thorough examination of study samples, including the relationship between ERP amplitudes, ADHD subtypes and symptoms, which could serve as an initial step in the aim to find the place of EEG/ERPs in diagnosis.

Limitations of our study include that a subset of patients received medication. However, in our subsidiary analyses we examined the impact of medication status on our findings, and found that results remained statistically significant after the adjustment for medication status. We should also note that although the two groups differed regarding task performance, the relatively good task performance in the ADHD group combined with the low probability of NoGo stimuli did not allow for separate analyses on correct and false NoGo trials.

## Conclusions

The current investigation revealed altered P1 amplitudes in adult ADHD patients, indicating that early sensory deficits are present in this patient population. This finding and its relationship to ADHD symptomatology are suggestive of bottom-up cognitive deficits in ADHD which are driven by impairments in early visual processing, and provide evidence that sensory processing problems are present at the neurophysiological level in this population. Since dysfunctional early stages of information processing can result in deficits in later stages, further research efforts should address these alterations more explicitly.

## Data Availability

The datasets used and/or analysed during the current study are available from the corresponding author on reasonable request.

## Abbreviations

ADHD: Attention-deficit/hyperactivity disorder
ERP: Event-related potential
EEG: Electro-encephalography
CPT: Continuous performance task
swLORETA: Standardized weighted low resolution electromagnetic tomography
SCL-90R: Symptom Checklist 90R
CAARS: Conners’ Adult ADHD Rating Scale
EOG: Electrooculogram
EMSE: Electromagnetic Source Signal Imaging
SAS: Statistical Analysis System
HLM: Hierarchical linear model
FDR: False Discovery Rate
ANCOVA: Analysis of covariance
LSMean: Least-Squares Mean
IFG: Inferior frontal gyrus
fMRI: Functional magnetic resonance imaging
